# Towards a Cheap and Easy Way to Monitor HIV/AIDS

**DOI:** 10.1371/journal.pmed.0020242

**Published:** 2005-07-19

It doesn't take a trained physician to know that a disease needs to be diagnosed to be treated. And it doesn't take an economist to know that a disease cannot be diagnosed if the required tools are unaffordable or impractical. The absence of early diagnosis and treatment is particularly problematic for infectious disease, where the lack of early treatment or isolation can result in an epidemic.

Given the technological requirements, diagnosis and monitoring of HIV infection is problematic in resource-poor areas. The advent of rapid tests for diagnosing HIV infection represents one part of the solution. Less clear is how patients diagnosed with HIV infection will be monitored, given the importance of CD4 cell counts. A decrease in CD4^+^ T lymphocytes—a critical immune cell infected by HIV—is one of the hallmarks of HIV disease, and CD4^+^ cell number is a key factor in determining disease progression and monitoring treatment. The methods for determining CD4^+^ cell numbers are technically complex, expensive, and not easily transportable. These factors severely limit the ability to monitor HIV disease in locations where resources, training, and mobility are limited.[Fig pmed-0020242-g001]

**Figure pmed-0020242-g001:**
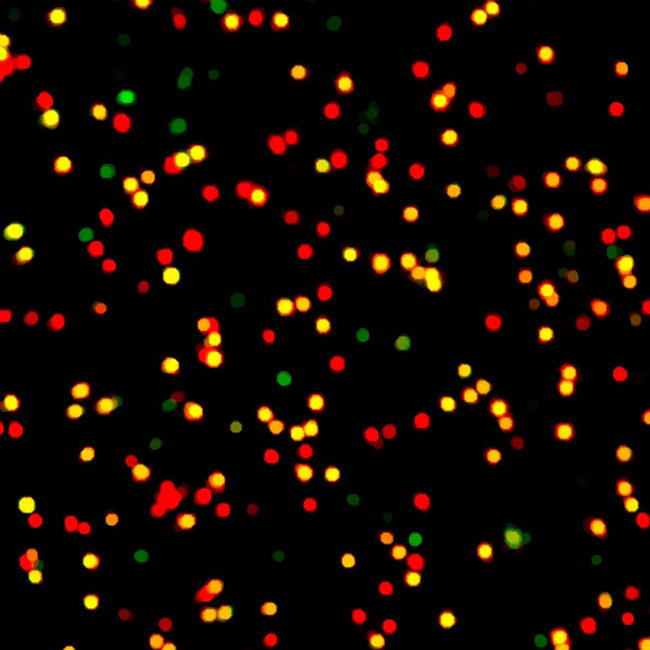
Digital image of whole blood obtained using the prototype device: CD4^+^ T cells shown in yellow, monocytes in green, CD8^+^ T cells in red

Lymphocytes are characterized by cell surface markers; thus, CD4^+^ lymphocytes express the CD4 marker on their surface. Antibody probes that specifically recognize this and other cell surface markers (such as CD8, which distinguishes that lymphocyte population from CD4^+^ lymphocytes, and CD3, which is a marker for all T lymphocytes) are used to count and differentiate various cell populations. By labeling cells with fluorescently tagged antibodies that recognize one or more cell surface molecules, the relative and absolute numbers of specific cells can be determined by a technique called flow cytometry. The labeled cells are passed through the flow cytometer, where the fluorescent probes are activated by lasers in a manner that can be read by specific detectors. The CD4^+^ cell number is directly correlated with the resultant fluorescent intensity and other light scatter properties. The problem is that flow cytometry requires costly reagents and substantial technical expertise—factors that limit its use in less developed areas.

Taking advantage of advances in microfluidics, digital imaging, and cell analysis, William Rodriguez and colleagues now report on a way to count CD4^+^ cells in a relatively quick, easy, and affordable manner. Small volumes of blood (an amount that could be obtained by a finger prick as opposed to drawing blood from a vein) are labeled as in flow cytometry, but with far less of the expensive reagents. Microfiltration allows the labeled CD4^+^ cells to be captured and separated from red blood cells, another simplification relative to flow cytometry. Digital images of the labeled cells, obtained by digital fluorescence microscopy, are then analyzed by newly developed software that can distinguish the CD4, CD8, and CD3 labels, thus allowing determination of absolute CD4^+^ counts, CD4^+^ percentages, and CD4^+^:CD8^+^ lymphocyte ratios.

Rodriguez et al. found that this new method was less accurate than flow cytometry for determining absolute CD4^+^ lymphocyte counts above 500 cells/mm3 (levels that are typically not relevant for monitoring HIV-infected individuals). But the method was as accurate as flow cytometry at clinically relevant levels of CD4+ cells for HIV-infected adult individuals. Although only a small number of pediatric patients were examined (and thus statistical significance could not be ascertained), the method appears to be also effective in determining CD4^+^ lymphocyte percentages in children.

The detection system used in the present report is a tabletop instrument that serves as a prototype for a fully portable handheld model, which is now under development. After some modest training, such a tool should allow a variety of health-care workers in remote areas to accurately analyze the CD4^+^ status of HIV-infected patients (the basis for treatment decisions) locally. In an accompanying Perspective discussing this new tool (DOI: 10.1371/journal.pmed.0020214), Zvi Bentwich argues that before it is ready for widespread use, several issues still need to be resolved, such as its final cost and its applicability to pediatric patients. “Despite these reservations,” he says, “the authors of this study should be commended for addressing an extremely important issue and developing this novel approach for counting CD4 in patients with HIV.”

